# Facial Neuralgia

**Published:** 1879-02

**Authors:** 


*Facial Neuralgia, and the Visceralgiæ, their Diagnosis and Treatment.* By J. Martin Kershaw, M. D.

This is Part I of a series on "Diseases of the Brain and Nervous System," and is devoted to the clinical history and symptoms of neuralgia, varieties, origin, &c.

In a section devoted to "Neuralgia of the Fifth Pair," family taint and constitutional defects, especially in children and growing young people are noticed, as well as Malarial Neuralgia and Migraine. The opinion of Dr. Spalding "that a large proportion of the cases of facial neuralgia located in the dental branches, are due to complete or approximate exposure of the dental pulp occasioned by decay" is cited, and a chronic form alluded to, having as a first cause dental decay, and which continues, in many instances, long after the cause is removed; and even extraction of the tooth does not afford relief in such cases. Constituional remedies are recommended, especially Aconite and Gelseminum.

Cases from Tomes, Wedl, Anstie, Erb, and others are given as evidence that cervico-brachial neuralgia can be traced to disease of the teeth, and that the removal of such teeth has at once relieved the neuralgic affection. Several wood cuts illustrate Part I.

